# Wearable Biosensors in the Workplace: Perceptions and Perspectives

**DOI:** 10.3389/fdgth.2022.800367

**Published:** 2022-07-04

**Authors:** Lauren C. Tindale, Derek Chiu, Nicole Minielly, Viorica Hrincu, Aline Talhouk, Judy Illes

**Affiliations:** ^1^Department of Obstetrics and Gynaecology, University of British Columbia, Vancouver, BC, Canada; ^2^British Columbia's Gynecological Cancer Research Program (OVCARE), BC Cancer, Vancouver, BC, Canada; ^3^Division of Neurology, Department of Medicine, University of British Columbia, Vancouver, BC, Canada

**Keywords:** wearable sensor, wearable electronic devices, occupational safety, biosensor, corporate ethics, workplace sensor, survey

## Abstract

**Objectives:**

Wearable body and brain sensors are permeating the consumer market and are increasingly being considered for workplace applications with the goal of promoting safety, productivity, health, and wellness. However, the monitoring of physiologic signals in real-time prompts concerns about benefit and risk, ownership of such digital data, data transfer privacy, and the discovery and disclosure of signals of possible health significance. Here we explore the perceptions and perspectives of employers and employees about key ethical considerations regarding the potential use of sensors in the workplace.

**Methods:**

We distributed a survey developed and refined based on key research questions and past literature to a wide range and size of industries in British Columbia, Canada. Both employers (potential Implementers) and employees (potential Users) were invited to participate.

**Results:**

We received 344 survey responses. Most responses were from construction, healthcare, education, government, and utilities sectors. Across genders, industries, and workplace sizes, we found a convergence of opinions on perceived benefit and concern between potential Implementers and potential Users regarding the motivation to use biosensors in the workplace. Potential Implementers and Users also agreed on issues pertaining to safety, privacy, disclosure of findings of possible medical significance, risks, data ownership, data sharing, and transfer of data between workplaces. The greatest variability between potential Users and Implementers pertained to data ownership.

**Conclusion:**

Strong agreement in the perception of biosensor use in the workplace between potential Implementers and Users reflects shared interest, motivation, and responsibility for their use. The use of sensors is rapidly increasing, and transparency about key use factors–both practical and ethical–is essential to maintain the current and desirable level of solidarity.

## Introduction

Workplace monitoring using wearable biosensors can be used to promote employee safety, productivity, health, and wellness (1, 2). Body sensors are growing in popularity, and brain sensors, although more recent, are also being implemented (3–5). Examples of intended workplace applications are fitness trackers that measure sitting time to decrease sedentary behavior in the workplace (6), wearable sensors that detect lumbar spine movement have been tested to prevent low back pain among workers (7), and posture sensors to identify work-related fatigue during surgery to reduce musculoskeletal disorders (8). Other sensors have the ability to detect concentration, energy expenditure, and emotional responses (9). For the brain, wearable sensors monitor employee alertness via electrical signals recorded from the scalp with the goal of increasing the safety of operators and drivers (3, 10).

Numerous wearable body and brain technologies are currently available in the open marketplace for personal use (3, 11), and an increased utilization is anticipated for the workforce (10). Alongside claims of benefit reside ethical and legal challenges such as data privacy, safety and protection, and the use of data for commercial gain, as well as the incidental identification of possible health findings (2, 4, 5, 12, 13). Other barriers to adopting health and safety monitoring technology include doubts about the reliability of and claims about the technology, lack of information about effectiveness, and even concerns about adequate IT support (14). Recently, the Organization for Economic Co-operation and Development (OECD) published recommendations for responsible neurotechnology innovation to help navigate these issues (15). The report includes recommendations for prioritizing safety assessment, promoting inclusivity enabling capacity of oversight and advisory bodies, safeguarding personal brain data, promoting cultures of stewardship and trust, and anticipating and monitoring potential unintended use and misuse.

Despite the expanding presence of wearables in western societies, there is limited academic research examining their use in the workplace (1), especially brain sensors, as well as employee acceptance of them (16, 17). This study is a start to fill this knowledge gap and provides evidence-based strategies for the ethical implementation and continued evolution of workplace sensors.

## Materials and Methods

### Survey Development

An online survey was designed and implemented using the Qualtrics survey platform (Qualtrics, Provo, UT). The survey was developed based on a background literature search and aimed to answer six key research questions: (1) What are employee views about participating in a workplace monitoring program that uses biosensors? (2) What motivates employers to implement a monitoring program that uses biosensors? (3) Do perceptions differ with type of biosensor, e.g., body or brain? (4) How will the safety, well-being of employees, and workplace culture be impacted by the introduction of wearable sensors? (5) What are the risks to employees and employers when data from wearable sensors are used to assess worker performance? and (6) Should the collected sensor data be transferred and follow a worker from workplace to workplace?

**Figure 1:**
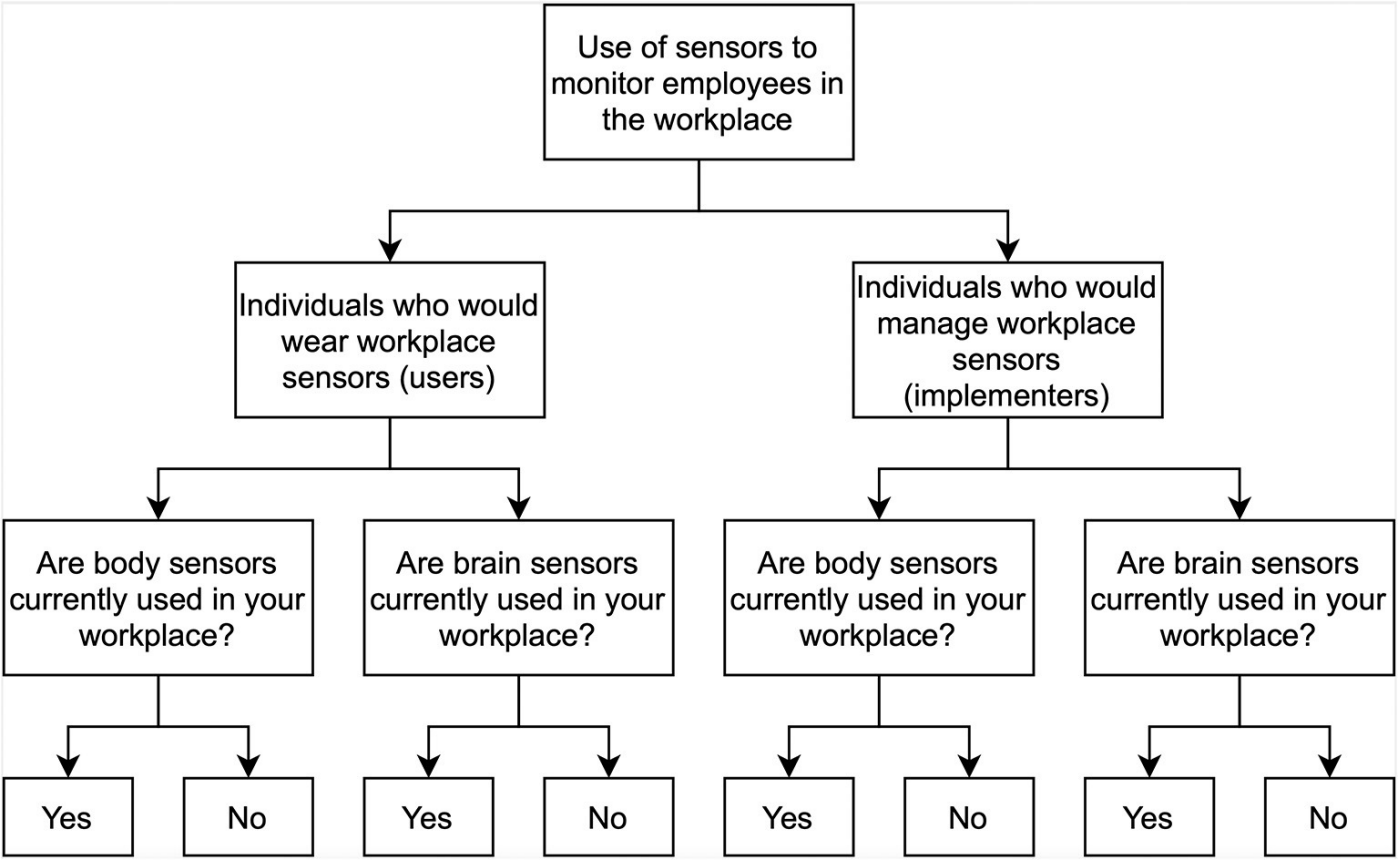
Survey branching logic.

The survey was divided into 6 sections: level of technological savviness, basic demographics, industry demographics, body sensor questions, brain sensor questions, and ethical questions. We implemented branching logic, with eight branches, to capture responses according to whether an individual was a potential User or Implementer and whether the person had prior experience with brain or body sensors (Figure 1). Using a crossover design, survey participants were randomly assigned to answer questions relating to body or brain sensor wearables first and then the complement.

Survey responses were analyzed using the statistical software R (18). Word clouds to capture free text responses were derived and used to enrich the quantitative results. Conceptual content analysis was used to assess themes from free-text responses (19).

### Recruitment

The survey was distributed across British Columbia (BC), Canada's third largest province with a population of about 5.1 million people, through social media and directly to 50,000 contractors associated with Technical Safety BC, the independent organization that regulates technical systems and equipment for BC. Respondents were required to self-identify as 18 years or older. Participation was voluntary and anonymous. Approval to administer the survey was obtained from the University of British Columbia's Behavioral Research Ethics Board. All respondents provided informed consent to participate and for their data to be used for the purpose of the research. The survey platform is compliant with the BC Freedom of Information and Protection of Privacy Act (FIPPA).

### Data Collection

The survey was launched in February 2020 but paused between March 2020 and March 2021 due to the COVID-19 pandemic. Data collection resumed through recruitment via Twitter, advertisements to BC audiences through Facebook and LinkedIn, and sent by email to members of two sponsoring BC safety associations. Participants followed a link to participate that redirected them to the survey. Following survey completion, participants were redirected to a second link where they could provide their email to be entered in a raffle for the chance to win one of five $100 Amazon gift cards. Role of the funding source

The funders of the study had no role in study design, data collection, data analysis, data interpretation, or writing of the report.

## Results

### Characteristics of Survey Responses

We collected 344 responses to the survey with >20% of the questions completed; 296 were completed entirely. Surveys with fewer than 20% of questions completed were considered spurious and not included in the analysis. Facebook ads were the most successful social media platform to gain respondents; Twitter posts and LinkedIn ads yielded minimal results. Facebook and LinkedIn tracked the click-through rate (CTR) of advertisements, which is the number of clicks divided by the number of times the ad was seen. Facebook ads had an average CTR of 2.22% and LinkedIn had a CTR of 0.57%. The survey took a mean of 14.17 min to complete (interquartile range [IQR] 12.71–15.63).

Upon descriptive inspection of the data and low Ns for brain data, we analyzed and reported the results combined with those for body sensors.

### Characteristics of Respondents

We received completed surveys from 53% male identifying and 43% female identifying respondents (Table 1). The majority were over 40 years (58%) (Table 1). Individuals who self-identified as potential *Users* constituted 59% of respondents. Forty one percent of respondents self-identified as potential *Implementers* responsible for implementing a sensors program and collecting data to monitor Users.

**Table 1:** Summary of survey variables by sensor users and sensor implementers.

**Variables**	**Total** ***n*** **(%)**	**Potential users** ***n*** **(%)**	**Potential implementers** ***n*** **(%)**
Gender	289	160	118
Male	152 (53)	69 (43)	75 (64)
Female	123 (43)	84 (52)	37 (31)
Undisclosed or other	14 (5)	7 (4)	6 (5)
Age	290	160	119
18–25	28 (10)	21 (13)	7 (6)
26–40	86 (30)	53 (33)	33 (28)
41–54	90 (31)	43 (27)	44 (37)
55–65	58 (20)	29 (18)	24 (20)
65+	21 (7)	12 (8)	7 (6)
Undisclosed	7 (2)	2 (1)	4 (3)
Industry	342	194	135
Construction	117 (34)	52 (27)	60 (40)
Healthcare	46 (13)	33 (17)	13 (10)
Education	37 (11)	29 (15)	7 (5)
Government	35 (10)	21 (11)	12 (9)
Utilities	34 (10)	19 (10)	14 (10)
Marketing, communications, sales	17 (5)	10 (5)	7 (5)
Manufacturing	13 (4)	8 (4)	5 (4)
Business, law, administration	11 (3)	10 (5)	0
Information Technology	10 (3)	2 (1)	8 (6)
Agriculture, forest, fishing	5 (1)	3 (2)	2 (1)
Transportation	4 (1)	3 (2)	1 (1)
Other	12 (4)	4 (2)	6 (4)
Size of workplace	341	194	135
Small (<20)	159 (47)	77 (40)	75 (56)
Medium (20–100)	71 (21)	45 (23)	25 (19)
Large (100+)	111 (33)	72 (37)	35 (26)
School completed	290	160	119
Post-secondary degree	110 (38)	65 (41)	41 (34)
Trades or vocational training	102 (35)	49 (31)	48 (40)
Graduate/professional degree	60 (21)	35 (22)	23 (19)
High school or equivalent	18 (6)	11 (7)	7 (6)
Less than high school	0	0	0
Body sensors used in workplace	315	180	135
No	300 (95)	171 (54)	129 (41)
Yes	15 (5)	9 (3)	6 (2)
Brain sensors used in workplace	317	183	134
No	310 (99)	180 (57)	130 (41)
Yes	7 (1)	3 (1)	4 (1)

Survey respondents were from construction, healthcare, education, government, utilities, and other sectors (Table 1). Respondents were distributed across small (<20 employees or self-employed), medium (20–100 employees), and large (100+ employees) sized workplaces (Table 1). Users and Implementers, genders, and workplace sizes were distributed across industries.

In response to the question “Are you tech savvy?” (scale: 0–10; 0 = not tech savvy, 10 = very tech savvy; IQR 6.29–6.70), overall self-reported responses yielded a mean of 6.50. In response to “Do you purchase the newest technology gadgets?,” using the same scale, respondents answered with a mean of 5.42 (IQR 5.20–5.64). 71/342 (50%) of respondents owned a body sensor; of this group 73% used it daily or weekly. By contrast, only 9/342 (3%) of respondents owned a brain sensor, but of this group 77% used it regularly.

### Body and Brain Sensors in the Workplace

Ninety five percent of respondents answered that body sensors are not currently being used to monitor employees in the workplace (Table 1). Ninety nine percent of respondents answered “No” to the same question about brain sensors. For those who answered No, all subsequent questions were posed in the hypothetical.

When asked about hypothetical or real motivations to use body and brain sensors in the workplace, top reasons among 130 respondents were, in order of ranking: employee health and wellness, employee safety, workplace productivity, workplace safety, and financial benefits. Free text responses included: “*I don't think it would help with trust at all”* and “*Mandatory wear would create a feeling on big brother watching me*.”

### Ethical Considerations

#### Benefits and Risks

**Figure 2:**
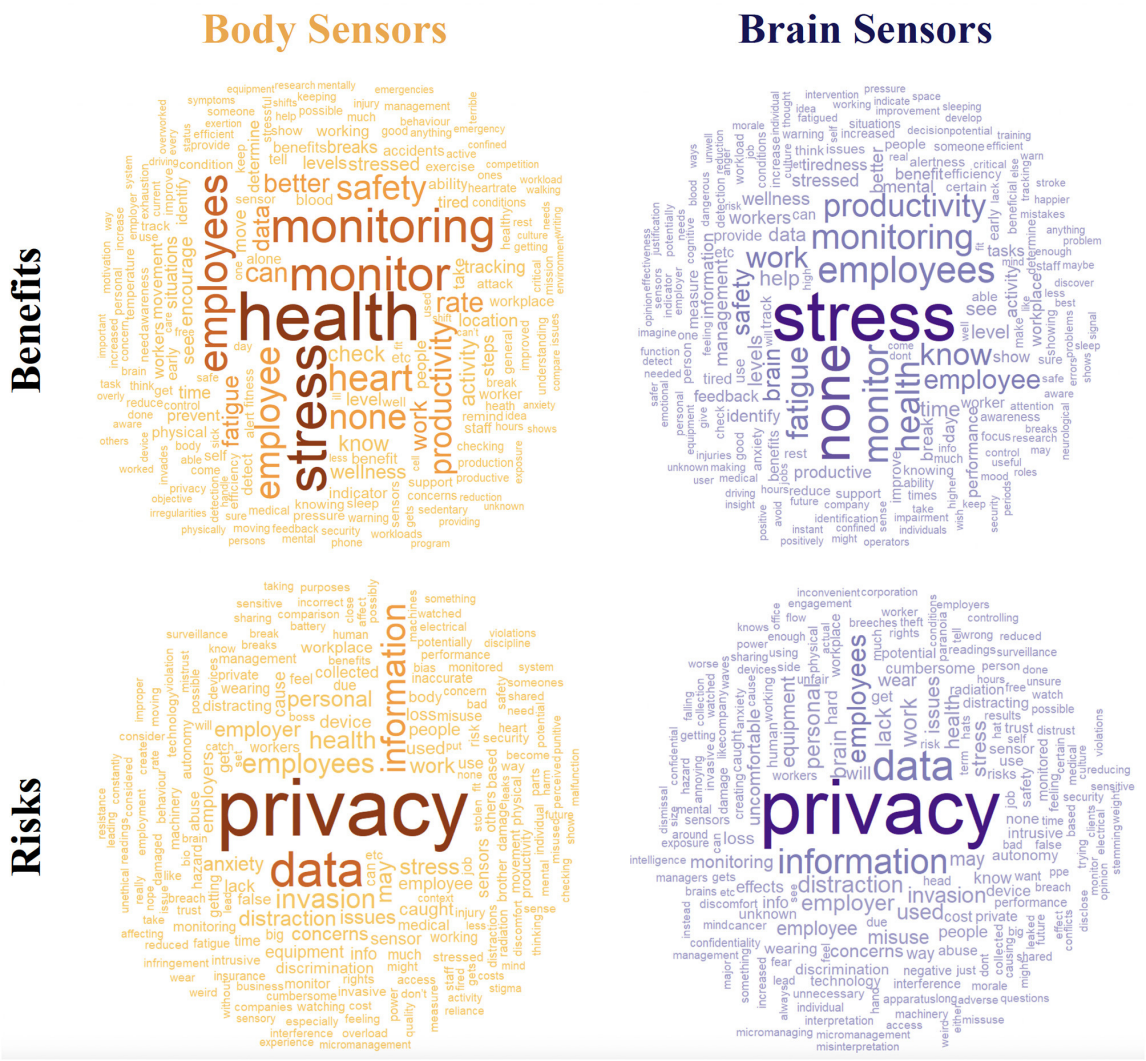
Word clouds showing the perceived benefits and risks of body and brain sensors expressed by both Users and Implementers.

**Figure 3:**
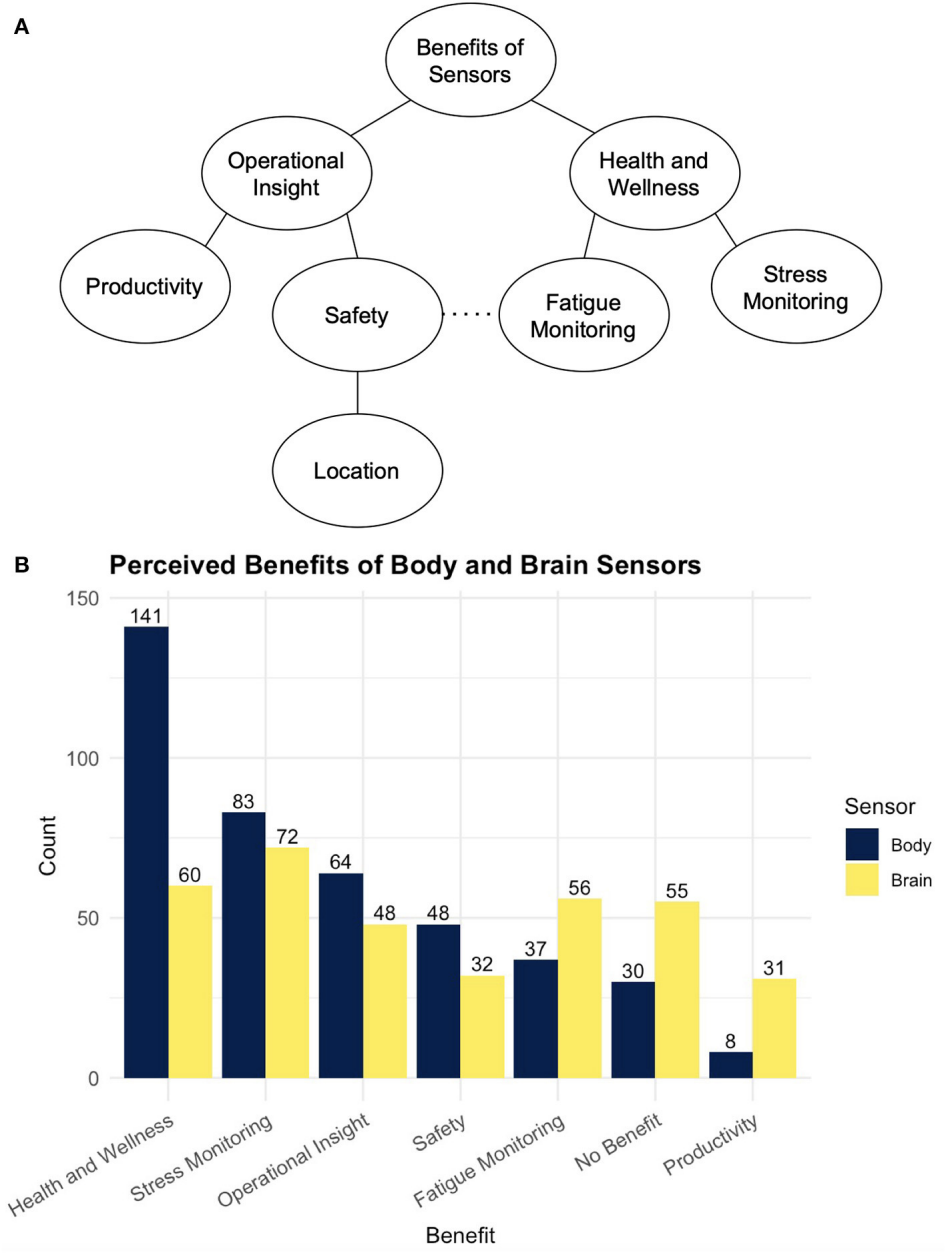
**(A)** Shows a tree diagram of the themes from free text responses about the perceived hypothetical benefits of using sensors in the workplace. **(B)** Shows the frequency at which each theme was mentioned body and brain sensors.

**Figure 4:**
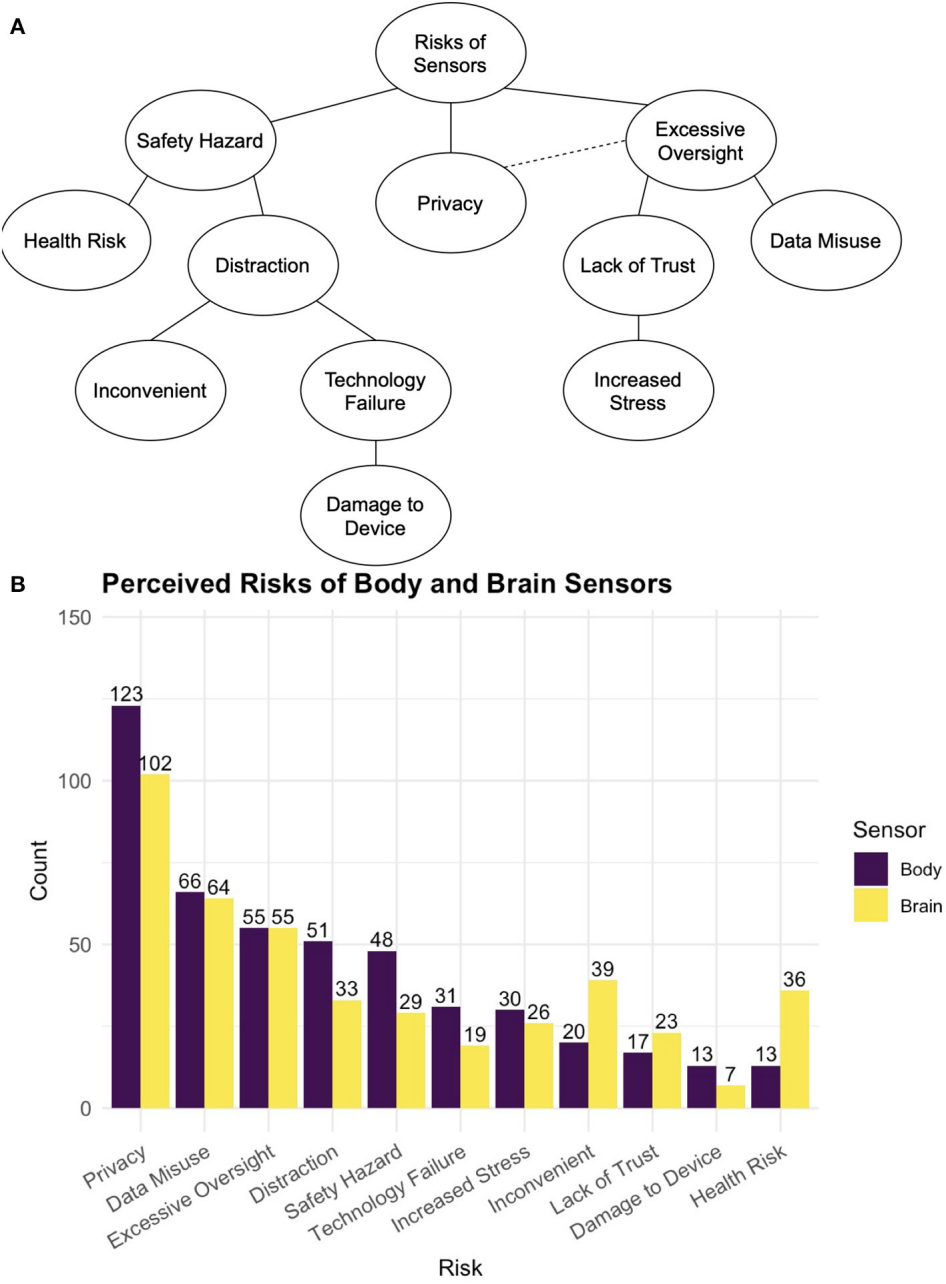
**(A)** Shows a tree diagram of the themes from free text responses about the perceived hypothetical risk of using sensors in the workplace. **(B)** Shows the frequency at which each theme was mentioned body and brain sensors.

Respondents were asked “What do you think are the benefits of using body sensors in your workplace? Please provide up to 3 that come to mind.” Equivalent questions were asked for risks and for brain sensors. Word clouds showing perceived benefits and risks to using body and brain sensors in the workplace are shown in Figure 2. Tree diagrams and frequency counts of the themes mentioned are shown in Figures 3, 4. For both body and brain sensors, employee health monitoring dominated perceived benefits; privacy dominated perceived risks. Body sensors had more perceived health and wellness benefits, while brain sensors were perceived to be more useful for fatigue monitoring and productivity. Brain sensors were also more likely to be perceived to have no benefit compared to body sensors. The largest perceived risks for both body and brain sensors were privacy, data misuse, and excessive oversight. Body sensors were more likely to be perceived as a potential distraction (e.g., “*focused on sensor, not on job”)* or safety hazard (e.g., “*getting caught in machinery*”). Brain sensors were more likely to be perceived inconvenient (e.g., “*uncomfortable*,” “*cumbersome*”) or a health risk (e.g., “*Potential low level radiation risks causing long term health issues*”).

#### Data Ownership and Transfer

**Figure 5:**
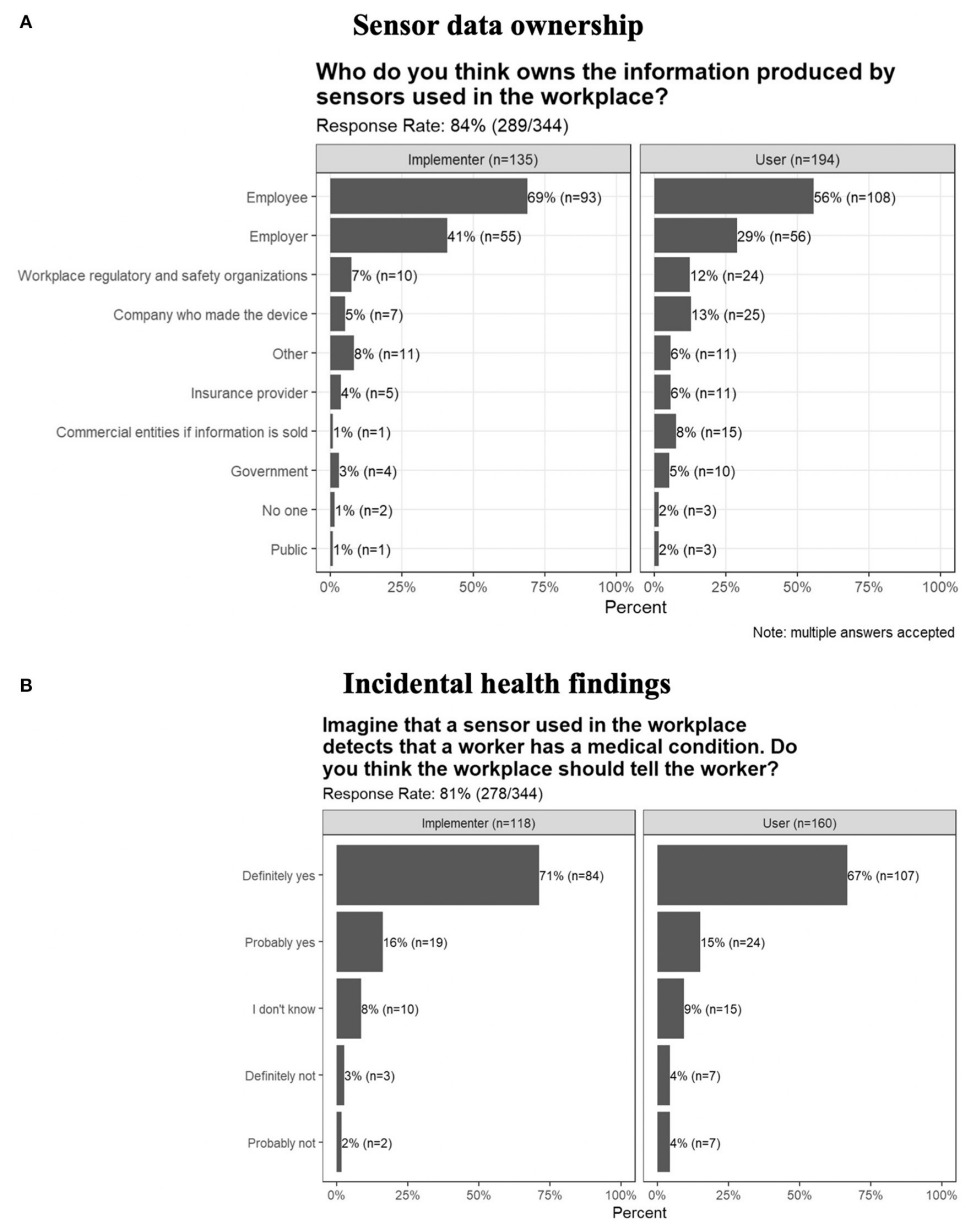
Opinions on **(A)** the sharing of incidental health findings, and **(B)** sensor data ownership by Users and Implementers.

In response to the question “Who do you think owns the information produced by sensors used in the workplace?,” where multiple answers were accepted, the majority of respondents (209/289; 72%) answered that it is the employee. Thirty eight percent answered that it is the employer, 12% answered that it is the workplace regulatory and safety organizations, and 11% answered that it is the company who made the device. Similar answers were seen when separated by gender, industry, and workplace size. There was evidence of a difference in proportion between potential Users and Implementers when the responses were collapsed into Employee, Employer, and All Other Responses (Pearson χ^2^ = 12.3; *p* = 0.002). Users were less likely to answer Employee (56%) compared to Implementers (69%), and answers were more varied than those from Implementers (Figure 5A).

In response to the question “If an employee moves to a new workplace, do you agree that their data can be transferred to the new employer?,” the majority of respondents (235/287; 82%) answered “No.” Similar distributions in answers were seen when separated by gender, User, Implementer, industry, and workplace size.

#### Communication, Disclosure and Consent

**Figure 6:**
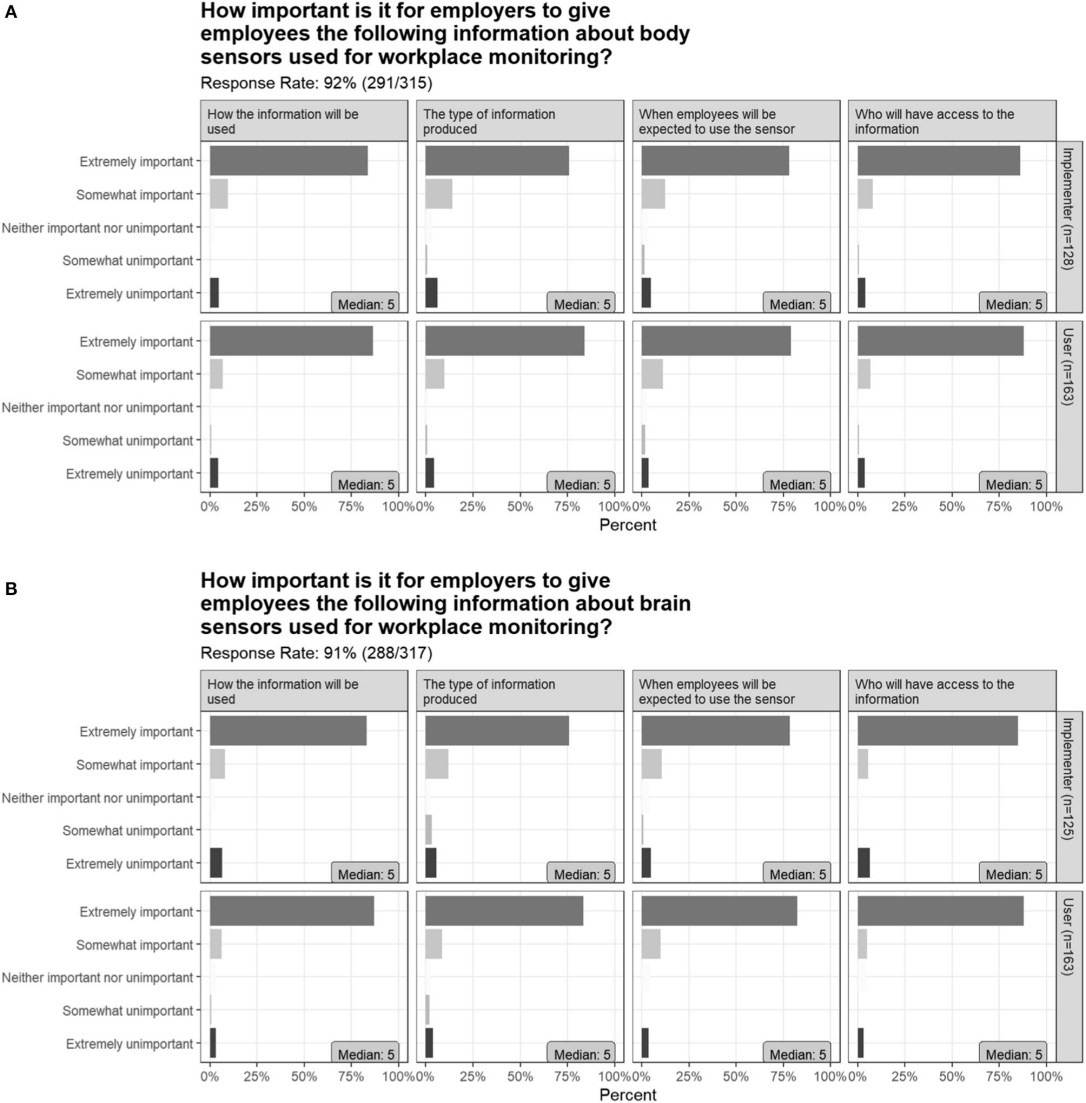
Survey responses about the importance of employers sharing workplace brain sensor data with employees.

When asked “How important is it for employers to give employees information about sensors used for workplace monitoring” with regards to: how the information will be used, the type of information produced, when employees will be expected to use the sensor, and who will have access to the information, in all cases >75% of respondents indicated “Extremely important.” This was true for both potential Users and Implementers, for both body and brain sensors (Figure 6).

#### Data Sharing

**Figure 7:**
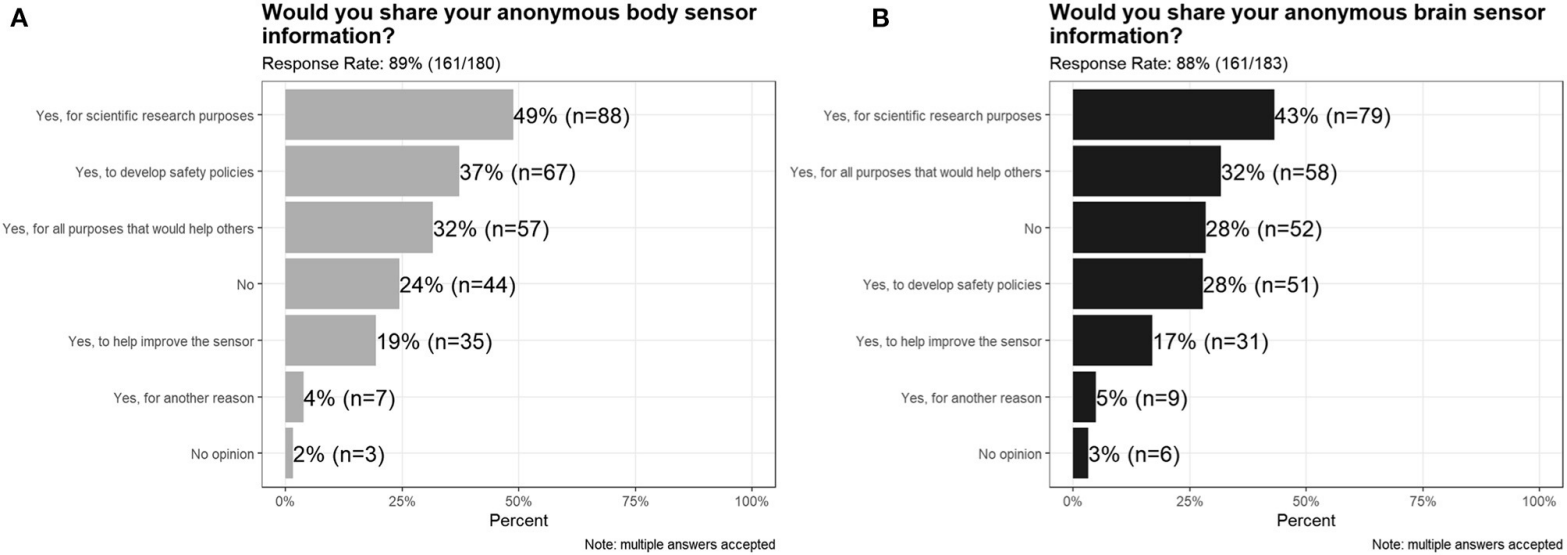
Survey responses to Would you share your anonymous **(A)** body sensor information and **(B)** brain sensor information.

The majority of survey respondents indicated that they would be willing to share their biosensor data anonymously for scientific purposes, to improve the sensor, or to develop safety policies, with only 24% of respondents saying that they would not share body sensor data, and 28% of respondents saying that they would not share brain sensor data (Figure 7). Free text answers included: “*To determine negative ramifications due to misuse of these devices,” “Work productivity, billing accuracy,” “To learn how to improve personal brain function in the future,” “We have seen the great benefit of Fitbit/Apple and other sensors to help individuals to align their health goals*,” and “*Self-awareness*.”

#### Signals of Possible Health Significance

In response to the question “Imagine that a sensor used in the workplace detects that a worker may have a medical condition. Do you think the workplace should tell the worker?,” the majority (191/278; 69%) answered “Definitely yes,” while other respondents were less sure, answering, “Probably yes” (15%), “I don't know” (9%), and “Definitely or probably not” (7%). Similar distributions in answers were seen when separated by gender, User/Implementer (Figure 5B), industry, and workplace size.

## Discussion

Perceptions and perspectives on body and brain sensors are convergent today among a large sample of regionally co-located potential User and Implementers and across genders, industries, and workplace sizes. Employee safety and wellness are the primary motivators to use sensors. Privacy of the digital data and excessive oversight are the primary considerations for risk. Communication, sharing, and transfer of data from workplace to workplace are more nuanced ethics variables of common prominence. Disclosure of findings of possible health significance is a must.

Answers to questions about sensor data ownership were the most variable. Potential User responses suggest that they are less sure about who owns the data and responded less uniformly that it should be the employee, compared to Implementers. Trust may be a key factor that contributes to this variability (20). Nonetheless, the results suggest that people are generally open to the idea of using biosensors in the workplace, and also being part of the process to improve them. Free text responses revealed that people were interested in learning how to gain personal betterment out of using biosensors, as well as monitoring for possible unethical uses of the data. Overall, the findings corroborate the OECD recommendations for responsible neurotechnology innovation (15), in particular pertaining to safety, safeguarding personal data, promote cultures of stewardship, and anticipating and monitoring potential misuses.

Although both employers and employees recognize the potential benefits of wearable biosensors, and many have had positive experiences with similar technologies using personal devices (Fitbits, Garmins, Apple watches), there are still major concerns about privacy, potential for data misuse, and excessive oversight in a workplace setting. Multiple survey respondents indicated their concerns citing a fear of moving toward an “Orwellian” and “Big Brother” type of surveillance.

Instances of companies using self-tracked app data where employees received incentives for providing and tracking personal health information have been previously reported (21); for example, some companies offer to pay for fertility-tracking apps to collect detailed information about their fertility and pregnancies for the purpose of helping companies minimize health-care spending and plan for medical problems. Some employees appreciated such health promotion initiatives, but others felt that there is too much potential for misuse with the intimate level of personal information asked for. It was observed however, that when introducing new voluntary health tracking initiatives, employees were initially hesitant but that their sensitivity decreased over time and they grew to see the initiatives as a benefit.

With the COVID-19 pandemic and the shift to remote work, companies may become more interested in monitoring productivity and health, particularly mental health. Remote working may also shift employees' perceptions of biosensors as workers become accustomed to using more technology to conduct their daily work. The survey was conducted both before and after the start of the pandemic, and could potentially be influenced by this shift in perspective.

### Recommendations

A rigorous ethical approach to the use and evolution of digital data from sensors in the workplace requires solidarity in understanding, expectations, and process between employers and employees, as well as greater involvement of regulators. As there is good agreement in perceptions between potential Users and Implementers, we propose a set of responsibilities that are mutually straightforward and easy to adopt. We describe the proposed responsibilities of employers, employees, and regulators and policy makers in Table 2. While a survey of regulators and policy-makers was beyond the scope of the present work, we extrapolate from the findings immediate roles for them to play as well. Key responsibilities involve disseminating evidence from empirical studies, interpreting them for industry, maintaining up-to-date guidance, and providing greater clarity than ever before about the space where unregulated wellness products meet regulated health products (4, 5).

**Table 2:** Recommendations for the responsibilities of employers, employees, and regulators/policy-makers.

**Employer responsibilities**
• Provide open and transparent information about the use of sensor data in the workplace. • Communicate intentions, both how and why sensors are being introduced or updated. • Outline clear expectations. • Explicitly obtain informed consent. • Ensure privacy protections. • Maintain safe storage. • Proactively manage access to data by third parties. • Have clear policies on findings of possible health significance. • Disclose changes in policy as they un-fold.
**Employee responsibilities**
• Understand company policies with regard to sensor data collection and use. • Understand processes involving in data protection, privacy, and transfer. • Provide or decline consent. • Report undisclosed or unexpected uses.
**Regulator and policy-maker responsibilities**
• Disseminate and make evidence from empirical studies on sensors in the workplace readily available to stakeholders. • Provide and communicate interpretation of findings. • Provide and maintain up-to-date guidance. • Implement regulations for wellness products that have the potential for health impact.

### Limitations

The small number of respondents from the individual industries prevented a cross-sectoral analysis. It is also possible that people in certain high-risk jobs such as fire fighters may perceive safety benefits from sensors as particularly life-saving and valuable, whereas people in low-risk jobs such as office workers may not attribute the same value to sensor data that measures sedentariness. In addition, given the mixed methods approach to recruitment (mailing lists, social media) the calculation of a response rate is not possible. We recognize that the individuals who chose to respond to the survey may have biases associated with a volunteer effect or had preconceived bias about biosensors that influenced their decision to participate in the study, and that self-perceptions of tech-savviness may vary.

## Conclusion

In order to successfully implement workplace biosensors, it will be crucial to gain the confidence of employees. The data suggest that many workers are already vary of personal monitoring biotechnology and increased surveillance. It will be the responsivity of workplaces and governing bodies to earn the trust of workers through clear policies, company-wide transparency, and ethical monitoring programs.

We conclude by suggesting that explicit and conscious attention to ethical and social responsibility in the context of monitoring signals from the human body and brain is not only feasible but a moral imperative. Western society is at a crucial point where upcoming successes and failures of sensor implementation will set the tone for public trust in the workplace environment—whether it is in-person or virtual—for years to come.

## Data Availability Statement

The raw data supporting the conclusions of this article will be made available by the authors, without undue reservation.

## Ethics Statement

The studies involving human participants were reviewed and approved by University of British Columbia's Behavioral Research Ethics Board. The patients/participants provided their written informed consent to participate in this study.

## Author Contributions

LT contributed to the data collection, data interpretation, and writing of the manuscript. DC contributed to methodological design and data analysis. NM and VH contributed to the methodological design and data collection. AT and JI contributed to the conceptualization of the study, methodological design, and data analysis and interpretation. All authors read and reviewed the final manuscript.

## Funding

The funding for this study was provided by Technical Safety BC and WorkSafe BC. The funding source had no role in the design, practice or analysis of this study.

## Conflict of Interest

The authors declare that the research was conducted in the absence of any commercial or financial relationships that could be construed as a potential conflict of interest.

## Publisher's Note

All claims expressed in this article are solely those of the authors and do not necessarily represent those of their affiliated organizations, or those of the publisher, the editors and the reviewers. Any product that may be evaluated in this article, or claim that may be made by its manufacturer, is not guaranteed or endorsed by the publisher.
